# Recent Advances in Kinase Drug Discovery Part I: The Editors’ Take

**DOI:** 10.3390/ijms22147560

**Published:** 2021-07-15

**Authors:** Julie A. Tucker, Mathew P. Martin

**Affiliations:** 1 Department of Biology, York Biomedical Research Institute, University of York, Heslington, York YO10 5DD, UK; 2 Cancer Research UK Newcastle Drug Discovery Unit, Newcastle University Centre for Cancer, Translational and Clinical Research Institute, Faculty of Medical Sciences, Newcastle University, Paul O’Gorman Building, Newcastle upon Tyne NE2 4HH, UK

**Figure 1:**
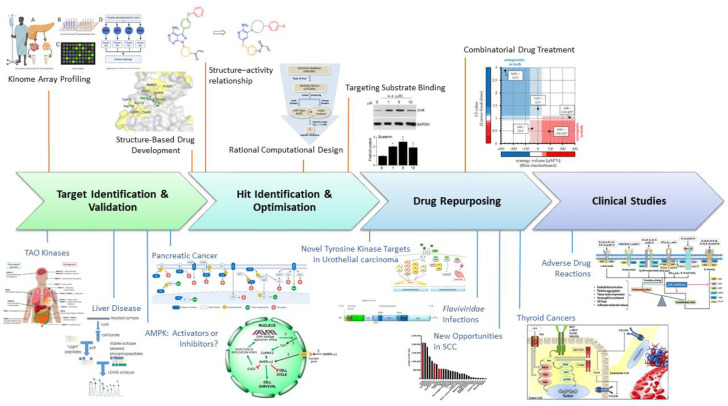
Recent advances in kinase drug discovery span the drug discovery pipeline from target identification through to pharmacovigilance. These advances are illustrated in six primary research articles covering kinome array profiling [4], structure-guided drug development [5,6], rational computational design [7], targeting the protein/peptide substrate binding site [8] and combinatorial drug treatment [9]; and nine topical reviews on TAO kinases [10], liver disease [11], AMPK [3], pancreatic cancer [12], urothelial carcinoma [13], *Flaviviridae* infections [14], squamous cell carcinoma [15], thyroid cancer [16] and adverse reactions to JAK inhibitors [17]. The indicated figures from special issue papers have been re-used with permission from the authors.

This special issue on Advances in Kinase Drug Discovery provides a selection of research articles and topical reviews covering all aspects of drug discovery targeting the phosphotransferase enzyme family. Recent technological advances across the drug discovery pipeline have enabled exciting progress in the area of kinase drug discovery (Figure 1). Sixty-nine protein kinase inhibitors had been approved for clinical use to 30 June 2021, including six in 2021, with many more under clinical investigation [1,2]. However, despite this panoply of approved drugs, there remains a significant unmet need in many disease areas.

Key to the achievement of a successful outcome in any drug discovery programme is a deep understanding of the underlying physiology and pathology of disease. The drug discovery process begins with target identification and validation (Figure 1) to which such understanding is vital. Our contributors address this stage in various ways.

Russell and Hardie [3] provide a comprehensive and scholarly overview of current knowledge of the biology and regulation of AMP-activated protein kinase (AMPK), which can act as both a tumour suppressor and an oncogene depending on cellular context. They critically review the available pharmacological modulators of AMPK, for which several classes of activators have been described, whilst comparatively little work has, to date, been published on inhibitors. Finally, they present a cogent argument for the use of AMPK activators in cancer prevention, and for the development of specific AMPK inhibitors for the treatment of established cancers either alone or, as sensitisers to DNA damage, in combination with cytotoxic agents. In particular, they note the potential role of AMPK-α1 specific inhibitors in personalised therapy for those cancers with elevated levels of the α1 isoform, and they encourage the field to discover and develop such inhibitors.

Five of our contributors focus their articles on the exciting potential for drug repurposing that is provided by the extant pharmacopeia, with an emphasis on hard-to-treat cancers and viral infections. The identification of novel uses for approved drugs and/or compounds with proven safety profiles can significantly accelerate the drug discovery process, bypassing the time-consuming hit-to-lead and preclinical stages and allowing a swift transition to the latter stages of clinical trials (Figure 1).

Torres-Jiménez and co-authors [13] provide an inclusive review of current clinical investigation of novel, molecularly targeted agents in urothethelial carcinoma, with a particular focus on advanced disease and the potential for tyrosine kinase inhibitors (TKIs). Despite advances in the molecular understanding of dysregulated pathways and increased access to appropriate patient-specific genetic signatures, many agents showing promise in pre-clinical studies and in other indications (including multiple EGFR inhibitors) have failed to show clinical benefit in the bladder cancer setting, whilst others (for example certain FGFR inhibitors) have improved response rates but remain a long way from being curative. Further research to understand why drugs that have worked in other settings are less effective in bladder cancers, how best to utilise those that are showing promise and to investigate newly identified pathways for their clinical utility is required.

Bensen and Brognard [15] summarise both currently approved and investigational treatment options for squamous cell carcinoma (SCC). They then go on to review the potential for development of novel treatments targeting alterations in the SCC kinome, both through the re-purposing of approved drugs and the discovery of novel chemical matter.

Lorusso and co-authors [16] provide a comprehensive review of the current clinical landscape in thyroid cancer, highlighting the proven and emerging roles for targeted kinase inhibitors as systemic therapy in otherwise refractory forms of this disease.

Blázquez and Saiz [14] review the potential for modulators of host kinase activity to act as novel anti-virals in the treatment of *Flaviviridae* infection. Host-directed antivirals are predicted to exhibit several advantages over more traditional, direct anti-virals, including a reduced propensity to drive the evolution of viral resistance. Promising studies in cell lines and animal models of flaviviral diseases, which include the mosquito-borne human pathogens dengue, Japanese encephalitis, West Nile and Zika virus, suggest this is an area worthy of further investigation.

The potential of host-directed anti-virals is confirmed by Wild and co-authors [9], who use both cell-based and animal models to assess the potential effectiveness of three cyclin-dependent kinase (CDK) inhibitors (abemaciclib, LDC4297 and maribavir) in the treatment of human cytomegaloviral (HCMV) infection, both alone and in combination. Their data demonstrate the effectiveness of these compounds against human, as well as, in some cases, murine CMV and other members of the herpes virus family. They propose that the remarkable effectiveness of LDC4297 arises from its impact on the host kinase, CDK7. Further work will be required to understand the full details of its mechanism of action and to explore the true potential of these and other host-directed anti-virals in the treatment of resistant infections.

In addition to new ways to approach established targets, and novel uses for known drugs, novel targets can provide additional therapeutic opportunities. Fang, Chang and Hsiao [10] provide an overview of the thousand and one kinase (TAOK) family, whose members are now recognised to play an important role in both normal and dysregulated physiological processes. The development of specific TAOK inhibitors is only just beginning, and this will be an interesting area to watch in the future.

Target identification and validation are aided by the application of modern technologies that allow for the monitoring not just of protein levels, but protein activity. Such techniques look set to revolutionise this area, as exemplified in an article by Yu and co-authors [11], who make the case for the use of kinome profiling to expand and deepen our understanding of kinase activity in healthy compared to diseased liver tissue. They provide a useful summary of two widely applied methodologies; mass spectrometry and array-based techniques. A number of kinase inhibitors are currently under clinical study in liver disease and this review concludes by summarising their status and postulating new targets.

The real-world application of protein activity profiling is illustrated in an article by Creeden and co-workers [4], who use a combination of bioinformatic analyses to mine the results of kinome array profiling data generated using both pancreatic ductal adenocarcinoma (PDAC) cell lines and healthy pancreatic tissue. On the basis of their analyses, they propose potential novel, actionable kinase targets in PDAC, a disease with few treatment options and a dismal outcome. In their accompanying comprehensive review [12], these targets are set in the context of disease-relevant pathways, and their known roles in both pancreatic cells and the surrounding stroma are described and discussed. It remains to be seen, however, whether inhibition of any of these targets alone or in combination has an impact on cell growth and ultimately tumour progression, and validation of these target candidates will be an important next step. Interestingly, known targets of FDA-approved drugs for pancreatic cancer were not always amongst the top differentially active kinases identified in this study, and this may reflect the increasing diversity of sub-types in pancreatic cancer, pointing the way for more specifically targeted therapy. The methodology presented herein could be more widely applicable to the identification of new kinase targets in areas where viable therapy is lacking.

Once a valid target has been identified, the process of hit discovery and optimisation begins (Figure 1). Traditionally, kinase inhibitors have been targeted towards the ATP-binding site, and although this approach has successfully delivered many approved drugs, it is often complicated by issues of selectivity, competition with high concentrations of cellular ATP and the development of resistance. Novel approaches to kinase inhibition are sought after, including, for example, the targeting of allosteric pockets, substrate peptide-binding sites, specific conformational states and cysteine residues. Advances in structure-guided drug discovery and design methodologies have contributed significantly to the medicinal chemist’s ability to harness these novel approaches and are illustrated in three contributions on this topic.

Rippin and co-authors [8] use a pharmacophore-based approach to identify novel, small molecule, substrate-competitive inhibitors (SCIs) of glycogen synthase kinase 3β (GSK3β) from a virtual screen of commercially available compounds. After a round of analogue selection, their most promising compounds exhibited single-digit micromolar potency in an in vitro kinase assay, were competitive with peptide substrate whilst non-competitive with ATP, and showed selectivity over other kinases in a kinase panel. Remarkably, given the low potency and anionic nature of the compounds, this activity translated to specific inhibition of GSK3 in both neuronal cell lines and primary cells. The authors note that this may result from the relatively low abundance of cellular substrates compared to cellular ATP. The SCI approach exemplified here could have broad applicability not just against protein kinases, but potentially many other systems where peptide-binding sites have been identified as potential avenues for inhibitor design, and we await further developments with interest.

Lee et al. [6] report the structure-guided design, synthesis and preliminary characterisation of a novel series of Bruton’s tyrosine kinase (BTK) inhibitors that target the inactive DFG-out conformation of the kinase as well as carrying a Michael acceptor to target Cys481 at the mouth of the ATP-binding site. Their lead compound shows potential advantages in terms of kinase selectivity over currently available compounds, and encouragingly, reduced the growth of tumour xenografts in a mouse model of haematological malignancy. It will be interesting to see whether this series does indeed act via a covalent mechanism as designed, and whether its kinase selectivity can be further improved through additional rounds of design and synthesis.

Park et al. [5] report the crystal structure of the emerging target, tyrosine protein kinase Mer (MerTK), in complex with a Checkpoint (Chk) kinase inhibitor, AZD7762. Interestingly, the structural data and impact of AZD7762 on the auto-phosphorylation of MerTK in two lung cancer cell lines suggest that AZD7762 targets a partially inactive state of the kinase. The 10-fold drop-off in IC50 measured in the in vitro kinase assay in the presence of cellular (1 mM) ATP concentration, however, is more consistent with the behaviour of conventional ATP-competitive, type I kinase inhibitors. Comparison of the MerTK:AZD7762 complex crystal structure with that of MerTK in complex with other inhibitor chemotypes and with other members of the Tyro3, Axl and MerTK (TAM) kinase family suggests some avenues for chemical modification that might address issues of selectivity and potency.

Park and colleagues [7] apply a dual-track virtual screening approach to identify novel inhibitors of the clinically relevant, drug-resistant EGFR mutant, EGFR [DelE746-A750 T790M C797S], with selectivity over wild-type EGFR. They implement an improved scoring function, which takes better account of ligand hydration effects, to improve their hit selection process, and screen in parallel against a homology model of the EGFR mutant and a crystal structure of the wild-type protein. Three of the 26 compounds identified in the virtual screen show low micromolar activity and impressive selectivity in a biochemical assay against the mutant and wild-type proteins, and two of these were taken forward for synthetic optimisation, steered by comprehensive structure-guided modelling. Despite nanomolar potency and >5000-fold selectivity in an in vitro kinase assay, however, their lead compounds show disappointingly limited selectivity in a cell-based assay, and further optimisation will be required to both understand the reasons for this disconnect and identify compounds with improved selectivity in cells.

The drug discovery process does not end with regulatory approval; monitoring for adverse effects continues once a drug has reached the market (Figure 1). As an example of the importance of this ongoing monitoring, Kotyla and co-authors [17] review the literature linking cytokine signalling to pro-thrombotic signalling with a view to illuminating mechanistic links underlying the clinically observed increased risk of thromboembolism in patients treated with inhibitors of the Janus kinase (JAK) family (Jakinibs). Their task is complicated both by the lack of selectivity of the currently approved Jakinibs, and the complex network linking cytokines via their receptors to downstream JAK activity. Even though each cytokine signals through its own receptor, there are fewer JAKs (four) than receptors (more than 20), such that multiple cytokines signal through the same JAK or combination of JAKs. Ultimately, Kotyla et al. suggest that additional ‘real-world’ data, ideally arising from the treatment of patients with as-yet unidentified, selective Jakinibs will be required to fully disentangle the contributions of patient pre-disposition versus inhibition of specific JAKs to increased thromboembolic risk.

Limitations of space and time mean that many exciting advances, including the application of biophysical techniques to larger and more complex systems, novel cellular target engagement assays and the burgeoning area of protein degraders, as well as the therapeutic targeting of lipid, nucleotide and small molecule kinases, are missing from this special issue. Several articles in this special issue highlight the use of computational methods to tackle current challenges in target validation and compound design [4,7,8]. The increasing application of artificial intelligence within the drug discovery pipeline has the potential to further enhance our ability to ‘drug’ those kinases currently considered ‘difficult’, for example, due to a lack of structural information. We are hopeful that these lacunae will be addressed in future editions.
